# Systematic review and meta-analysis of HIV, HBV and HCV infection prevalence in Sudan

**DOI:** 10.1186/s12985-018-1060-1

**Published:** 2018-09-25

**Authors:** M. M. Badawi, M. S. Atif, Y. Y. Mustafa

**Affiliations:** 1 Medical Microbiology department, Faculty of Medical Laboratory Sciences, Elrazi University, Khartoum, Sudan; 2 Medical Microbiology department, Faculty of Medical Laboratory Sciences, University of Khartoum, Khartoum, Sudan

**Keywords:** Africa, Developing countries, Epidemiology, Middle East, Viral infections

## Abstract

Viral hepatitis constitutes a global health problem; previous studies have affirmed a considerable morbidity and mortality from both acute infections and chronic complications. On the other hand, Human Immunodeficiency Virus (HIV) infection is also of known burden. Determining prevalence measures of these viruses is crucial for establishing appropriate country specific strategies regarding prevention, diagnosis, and containment. This systematic review was aimed to provide pooled seroprevalence estimates of the three viruses in Sudan. Structured review of the literature was conducted to obtain relevant studies published in both national and international databases. After assessment of quality and bias in all proposed studies, 57 prevalence studies were included. Meta-analysis was conducted for all studies and subgroup analysis was also approached. The total sample size of participants in included studies providing HIV antibodies prevalence was 15,479. Based on information retrieved from these studies, HIV prevalence ranged from 0 to 18.3% among different study populations. However, pooled prevalence estimate for HIV antibodies was 1%. Kassala, Eastern Sudan was the most endemic State (4.18%). The HBV reported seroprevalence rates ranged from 5.1 up to 26.81% among different populations and the overall pooled prevalence was 12.07%. For HCV antibodies; 2.74% was determined to be the pooled prevalence. Khartoum State was the most endemic State of both HBV and HCV with seroprevalence of 12.69% and 6.78%, respectively.

Based on data reviewed and synthesized; there is no evidence for an HIV endemic in the general population of Sudan. However, both HBV and HCV seroprevalence rates are indicating otherwise. Reducing the overall burden of HIV, HBV and HCV infections will require new measures and national strategies and the recognition of the infections as one of the country’s priority issues.

**Electronic supplementary material:**

The online version of this article (10.1186/s12985-018-1060-1) contains supplementary material, which is available to authorized users.

## Background

Viral hepatitis constitutes a global health burden. Previous studies have affirmed a considerable morbidity and mortality from both acute infections and chronic complications including chronic hepatitis, cirrhosis, and hepatocellular carcinoma (HCC). More than one million people die each year from hepatitis C virus and hepatitis B virus infections. Chronic HBV infection affects over 350 million people worldwide while 150 million people have chronic HCV infection [1]. On the other hand, the global burden of Human Immunodeficiency Virus (HIV) in 2016 was 0.8% among adults, around 30% of them do not know that they are infected [2]. Co-infection of HBV/HCV and HIV is characterized by more rapid progression of liver diseases; including accelerated fibrosis, cirrhosis, and HCC [3]. These viruses share the main transmission routs as they are blood borne pathogens. Transmission occurs from patient to patient via unsafe sexual practices in HIV and HBV while it’s rare in HCV or vertically during pregnancy from mother to child. The viruses can be transmitted from patients to health care personnel via contaminated instruments or accidental needle-stick or sharp injuries [3–5]. Onwards transmission of these viruses could be related to several reasons including: missing opportunities for prevention, lack of awareness about their prevalence and prevention, misdiagnosis, absence of medical care and poor health outcomes in infected people [3–5].

Data exist concerning the epidemiology of HBV, HCV and HIV in several study populations in Sudan. Several studies have provided estimates of the prevalence measures of the three viruses among pregnant women, blood donors and hemodialysis patients as these populations have their impact in estimating the burden of both the wide prevalence among whole population and the prevalence among populations at risk. Chaabna and colleagues have recently (2016) published a systematic review and meta-analysis only for HCV prevalence in Sudan among other neighboring countries. However, although the included studies have been assessed for the risk of bias in their study, all studies were included in the quantitative analysis to estimate the pooled prevalence of the virus antibodies [6].

The aim of this study was to provide a systematic review and meta-analysis of the results of prevalence studies of the three viruses in different populations and in distinct geographical regions, which will help in determining the population distribution of the viruses and the association of the three infections - if any, contribute in planning national strategies for containment and awareness raising campaigns as well as designing specific preventive measures.

## Methods

### Search strategy

To identify relevant studies, a systematic review of the literature was conducted in the period from January to April 2017, studies published in 2017 were not included. The review was conducted in accordance with the PRISMA (Preferred Reporting Items for Systematic Reviews and Meta-Analyses) Statement [7] (Additional file 1: Table S1). A comprehensive search was conducted in PubMed, Embase, Google scholar, Scopus, Index Copernicus, DOAJ, EBSCO-CINAHL, Cochrane databases as well as Sudan Journal of Medical Sciences without specific time or language limits. The keywords used were Human Immunodeficiency Virus, Hepatitis B, Hepatitis C, Hepatitis B surface antigen, prevalence, Sudan, and similar terms such as HBV, HBsAg, HCV and HIV were also crossed. Moreover, to optimize our search, hand searches of reference lists of included articles were also performed.

### Study selection and data extraction

Two authors (BMM and AMS) independently assessed titles and abstracts for eligibility, and any disagreement was resolved through discussion. A copy of the full text was obtained for all research articles that were available and approved in principle to be included. Abstraction of data was in accordance of the task separation method; method and result sections in each study were separately abstracted in different occasions to reduce bias [8]. Moreover, data abstraction was conducted with no consideration of authors’ qualifications or expertise. Each research article was screened for all relevant information and recorded in the data extraction file (Microsoft Excel), as one article may report outcome of several prevalence studies; prevalence data of HIV, HBV or HCV for a defined population group, in a defined region, or combine two or more of them (co-infection prevalence) or provide specific prevalence in one infection under study as Occult Hepatitis B (OBI). Moreover, data from each method section was extracted using a predefined set of variables; study characteristics, type of participants, study population size, geographical region and the screening protocol used.

### Assessment of quality and risk of bias

Each included article was evaluated for its quality based on a framework for making a summary assessment of the risk of bias. The framework was developed specifically to assess the risk of selection bias, to determine the level of representativeness of the studied population and to judge the strength of the estimates provided by the included studies. Three different frameworks were proposed to accommodate the differences between seroprevalence studies with regard to different study populations. All studies conducted not-confirmed ICT screening protocol (i.e. seropositive result of a sample is determined when observing ICT positivity only without any further confirmation applying other ICT protocol or ELISA) was not included in the assessment of the quality and risk of bias and was immediately excluded. The only exception was studies that conducted in States other than Khartoum State, as very limited studies were available and in several cases only one study was found to represent the entire State, and the study characteristics of those articles are summarized and presented alongside the prevalence rates in (Additional file 1: Tables S2 & S3). Trim and Fill method was used to assess the risk of publication bias in the included studies [9]. Publication bias was assessed separately for research articles providing prevalence data for HIV antibodies, HBsAg and HCV antibodies.

### General population and populations at high risk

All authors - after comprehensive discussion - approved the following four domains to be considered as possible sources of selection bias in general population studies and studies toward specific populations at risk: age, gender, population coverage (i.e. the population covered by the sampling design in geographical manner) and sample size. Studies conducted in Female Sex Workers (FSX) were not assessed for gender bias. Points were given for representativeness or a lower risk of bias in each domain (Table 1). A total score for risk of bias was calculated by adding up the scores in all four domains, resulting in a score of between 0 and 7. The highest score indicates the lowest risk of bias, studies in the general population or those toward specific populations at risk with a score for risk of bias greater or equal to 3 (higher quality) were included in the review. All authors agreed that the four domains are the only sources of bias a reader can determine; inclusion of different age groups, inclusion of both sexes, conduction of the study in different regions/locations and more study participants were considered vital to reduce the selection bias to the minimum.

**Table 1:** Assessment of quality of general population studies and studies toward specific Populations at risk

Aspect	Score	Description
Age	0	Not determined
	0	Determined with clear bias; not representative to general/targeted population
	1	Determined with no clear bias; can be considered representative
Gender	0	Not determined
	0	Determined with clear bias
	1	Determined with no clear bias; can be considered representative
Coverage	1	Single centre/local
	2	Multi-centre/local
	3	Multi-centre/national
Sample size^a^	0	Less than 150 participants
	1	More than 150 and less than 1000 participants
	2	More than 1000 participants

^a^Thresholds were set based on authors opinion solely

### Blood donors and pregnant women

Two domains were considered possible sources of selection bias in studies providing prevalence rates among blood donors and pregnant women; population coverage and sample size. Points were given for representativeness or a lower risk of bias in each domain (Table 2). A total score for risk of bias was calculated by adding up the scores in the two domains, resulting in a score of between 0 and 5. Studies among blood donors or pregnant women with a score for risk of bias greater or equal to 2 were included in the review. The reason behind that gender and age was not considered as possible sources of selection bias in these studies is that blood donors are mainly males in Sudan, and both pregnant women females and blood donors males are mostly from a specific age interval.

**Table 2:** Assessment of quality of studies providing prevalence rates among blood donors and pregnant women

Aspect	Score	Description
Coverage	1	Single centre/local
	2	Multi-centre/local
	3	Multi-centre/national
Sample size^a^	0	Less than 150 participants
	1	More than 150 and less than 1000 participants
	2	More than 1000 participants

^a^Thresholds were set based on authors opinion solely

### Quantitative analysis

Meta-analysis was performed using Review Manager Software (Version 5.3). In studies where the Standard Error (SE) is not reported, the following formula was used to calculate it: SE = √p (1-p)/ n where p stands for Prevalence. The software automatically provided the Confidence Interval (CI) according to the calculated SE, if the CI is provided in a study; it was introduced accordingly. Freeman-Tukey double arc-sine transformation was used to stabilize the variance in extreme prevalence measure. The heterogeneity of each meta-analysis was assessed, the random effects was favored over the fixed effects model in all meta-analysis established as it sought suitable for determining prevalence estimates. We conducted subgroup analysis for studies conducted in the same State as well as studies published in the same year to provide pooled prevalence estimates according to geographical regions and publication period, respectively. Sensitivity analysis was also approached to determine the effect of studies conducted in populations at risk of infections on the overall pooled prevalence. Data were introduced into (Adobe Photoshop) and (SPSS) to illustrate geographical maps and trend of prevalence according to publication period, respectively.

## Results

### Studies included

**Fig. 1:**
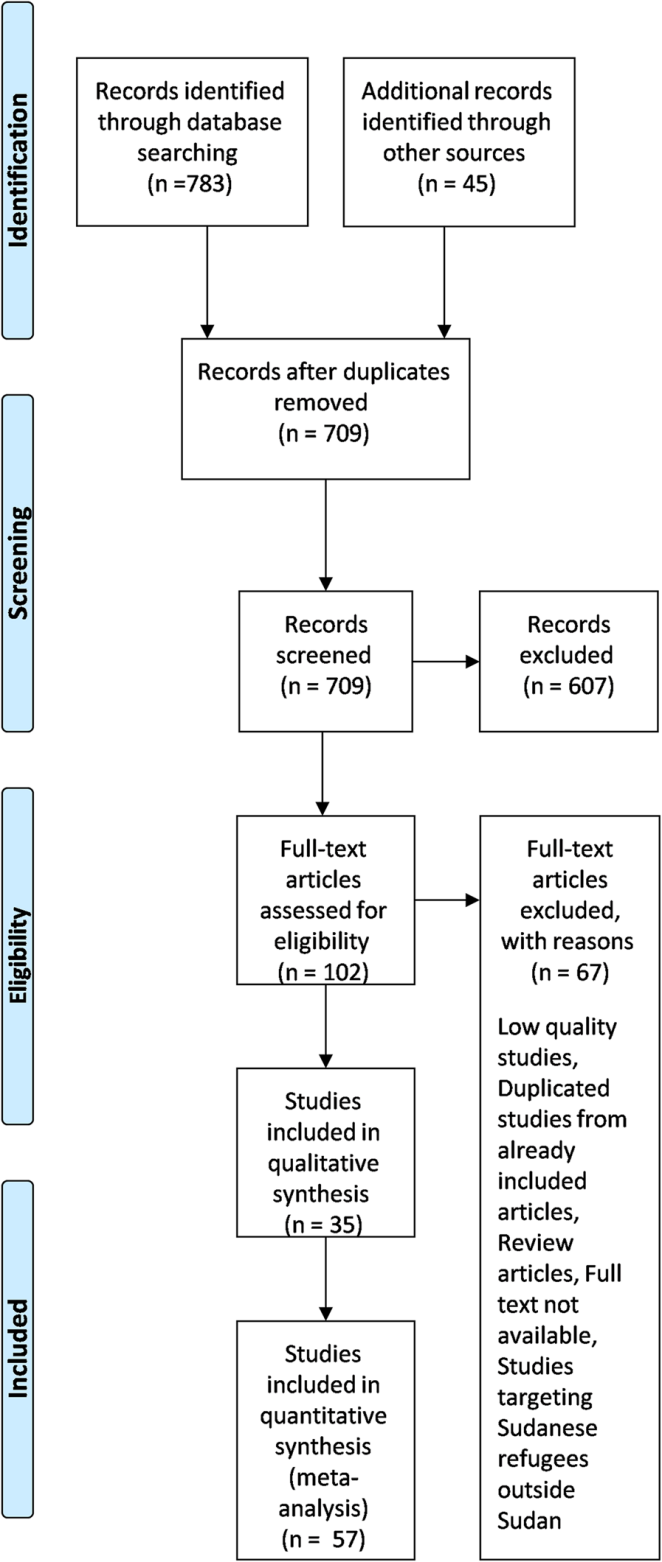
Literature search and selection of studies (PRISMA flow diagram)

A total of 709 articles were identified from the search strategy including hand searches of reference lists of included articles. From these, 674 articles were excluded. Thirty five articles met our inclusion criteria. The articles reported the prevalence of HIV antibodies and/or HBsAg and/or HCV antibodies and Occult Hepatitis B. (Fig. 1) illustrates the PRISMA flow diagram. A total of 57 prevalence studies were extracted from the articles. The included articles are depicted in (Additional file 1: Table S2).

### Study characteristics

The characteristics of the included studies are depicted in (Table 1), among which the oldest was published in 1997 while the most recent ones were published in 2016. Twelve research articles determining HIV antibodies prevalence were included. Furthermore, fourteen and nine articles concerned of prevalence measures of HBsAg and HCV antibodies were included, respectively. Publication bias assessment indicated no major asymmetry (data not shown).

### Overall national prevalence

**Fig. 2:**
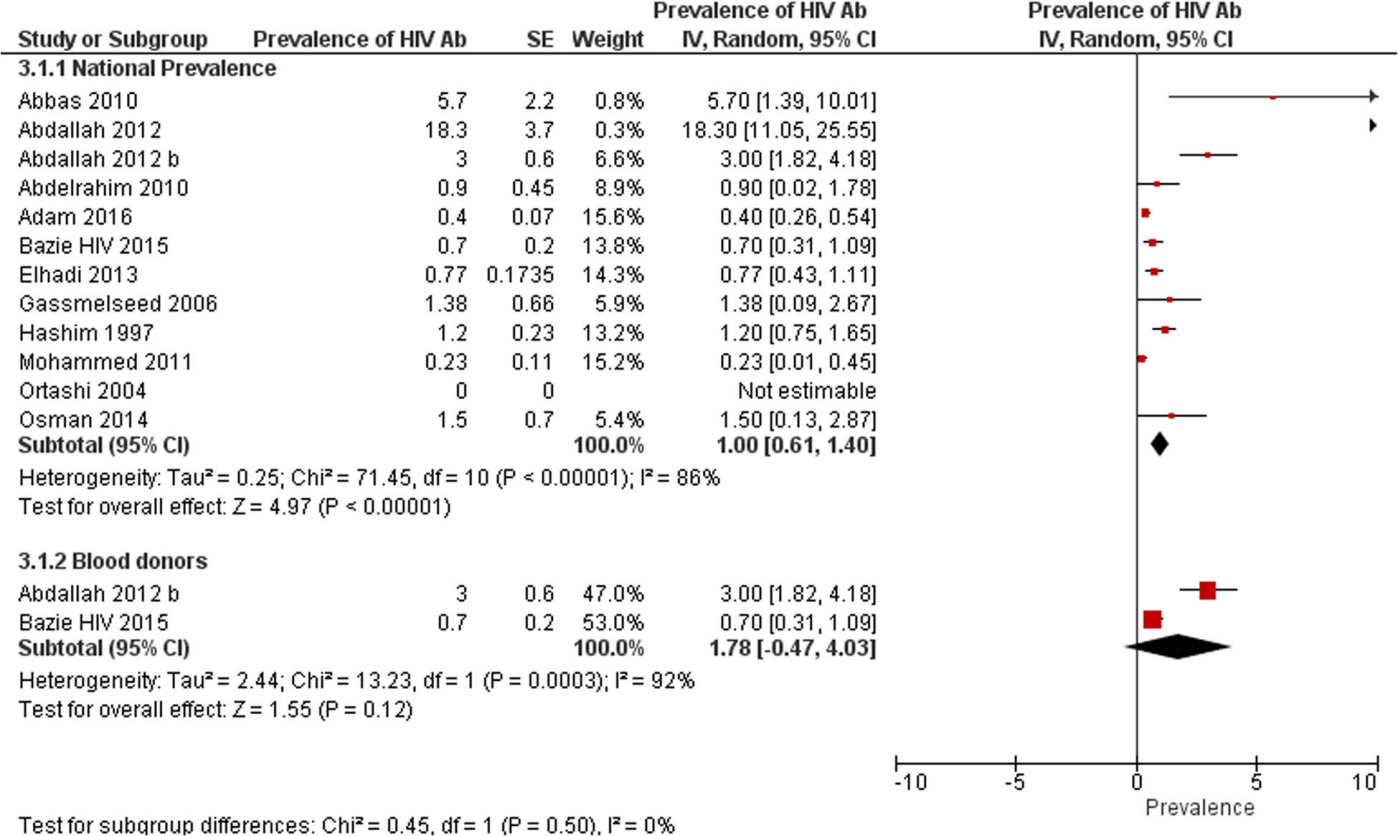
National prevalence of HIV antibodies and prevalence among blood donors from studies included in the review

A total of 12 eligible articles were found determining prevalence of HIV antibodies in Sudan [10–21]. The total sample size of participants was 15,479. Based on information retrieved from these studies; HIV prevalence ranged from 0 to 18.3% among different study populations, 75% of studies (9/12) reported a prevalence lower than 3% (0.7% [10], 1.3% [11], 1.5% [12], 0% [14], 0.4% [15], 0.3% [17], 0.9% [19], 0.7% [20] and 1.2% [21]), while 25% (3/12) of studies reported a prevalence equal or higher than 3% (3% [18], 5.7% [13], and 18.3% [16]). The result of meta-analysis; (Fig. 2) showed overall pooled prevalence of 1% (95% CI 0.61 to 1.40), the heterogeneity was I^2^ = 86%. Sensitivity analysis was 0.88% (95% CI 0.49 to 1.27). There was no included population-based study to be considered representative to the whole population, we were of no confidence to consider studies conducted on blood donors as population-based studies as no enough information was found to determine whether they were first time blood donors or they have donated previously (i.e. screened previously).

**Fig. 3:**
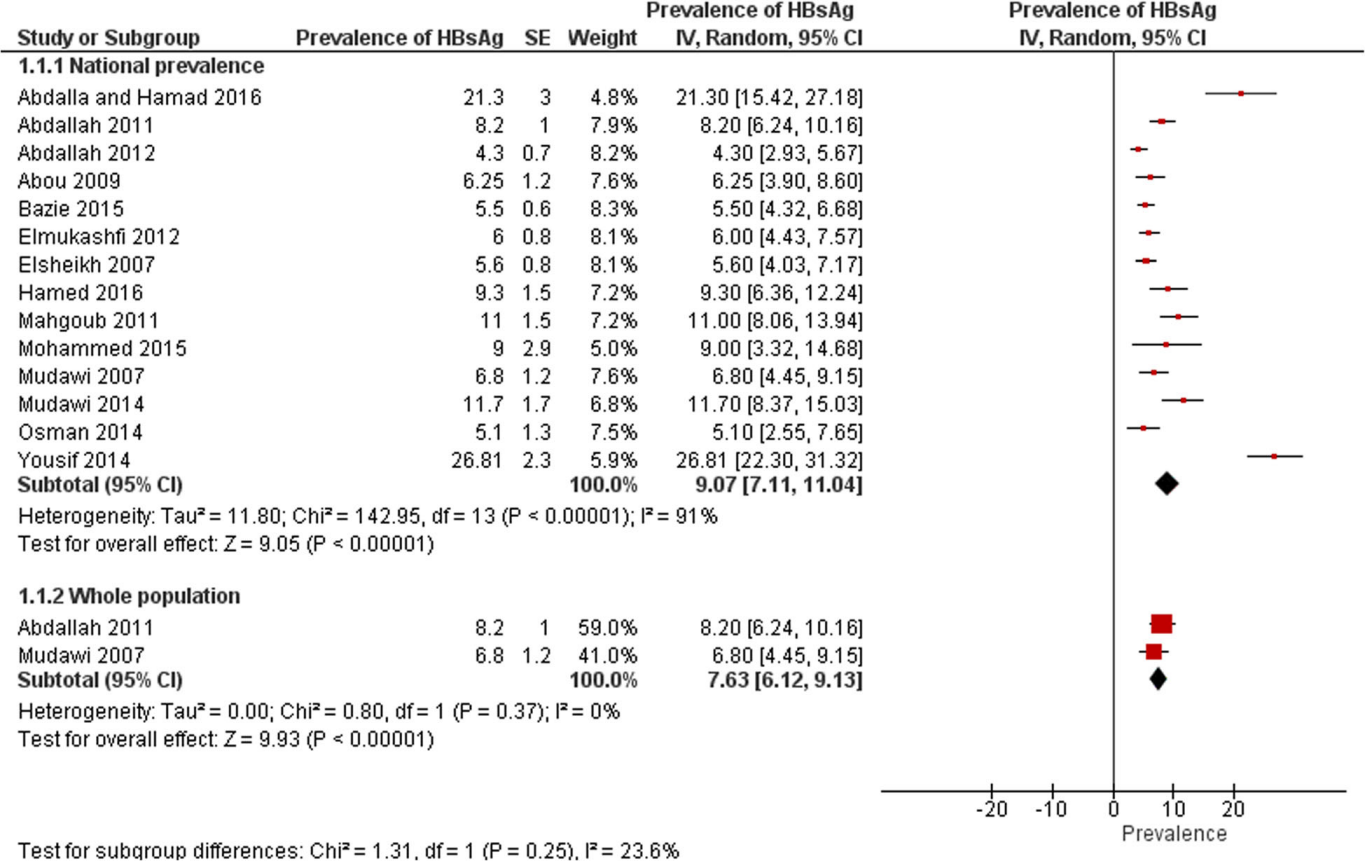
National prevalence of HBsAg and prevalence among whole population from studies included in the review

A total of fourteen eligible studies were found to determine prevalence of HBsAg in Sudan [12, 18, 20, 22–32]. The total sample size of all studies was 5848 participants. Among the 14 studies; the reported prevalence rates ranged from 5.1 up to 26.8% among different study populations. Half of studies 50% (7/14) reported a prevalence lower than 8% (5.1% [12], 4.3% [18], 5.5% [20], 6.25% [24], 6% [25], 5.6% [26] and 6.8 [30]), almost 36% (5/14) of the studies reported a prevalence rates higher than 8% (8.2% [23], 9.3% [27], 11% [28], 9% [29] and 11.7% [31]). Two studies (almost 14%) reported prevalence rates higher than 20% (21.3% [22] and 26.8% [32]). The pooled prevalence was 9.1% (95% CI 7.11 to 11.04), the heterogeneity was high (I^2^ = 91%) (Fig. 3). The pooled prevalence after conducting the sensitivity analysis was 6.6% (95% CI 5.27 to 7.99). Only two studies among the fourteen studies were concerned of the prevalence of HBsAg among general Sudanese population [23, 30]. Both studies was not conducted in Khartoum State, one study have been concerned with the population of Eastern Sudan as their study was established in Kassala State [23]. The other study was conducted in a village called Um Zukra in the Managil province, Gezira State of Central Sudan [30]. It is to be noted that the later study is conducted in *Schistosoma* endemic area; however, authors concluded no significant association between HBV infection and history of *Schistosoma* infection. Furthermore, and to be more confident in classifying the study’s population as general population; the same population was found to be tested in another study earlier, and they also determined lack of association between the two infections [33]. The study of Abdalla and colleagues [23] determined the prevalence of HBsAg as (8.2%) among all study participants, in the other study conducted in the Managil province the prevalence was determined as (6.9%). The pooled prevalence was 7.6% (95% CI 6.12 to 9.13), the heterogeneity was I^2^ = 0%.

**Fig. 4:**
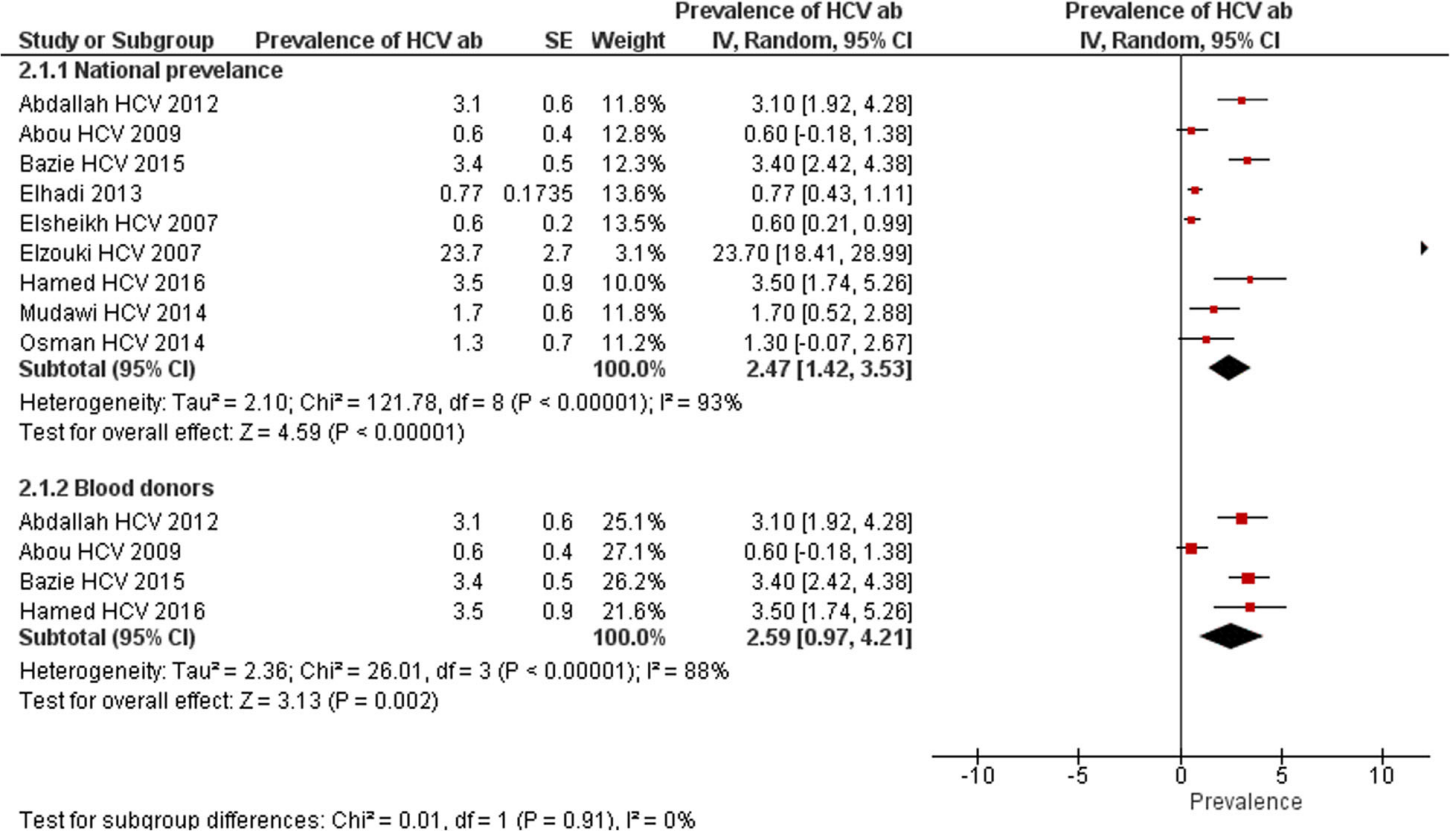
National prevalence of HCV antibodies and prevalence among blood donors from studies included in the review

Regarding HCV; a total of nine research articles were identified establishing the prevalence of HCV antibodies in Sudan [10, 12, 18, 20, 24, 26, 27, 31, 34]. The total sample size of all studies was 8643 participants. The reported prevalence rates among the nine studies ranged from 0.6 up to 23.7% among different study populations. 4/9 (44%) estimated rate of prevalence above 2% (3.1% [18], 3.4% [20], 3.5% [27] and 23.7% [34]). The overall pooled prevalence was 2.5% (95% CI 1.42 to 3.53), the heterogeneity was high (I^2^ = 93%) (Fig. 4). The pooled prevalence after conducting the sensitivity analysis was 2% (95% CI 0.85 to 3.13). No included study has been found providing prevalence information in normal healthy population.

### Prevalence among blood donors

Only two included studies determined the prevalence of HIV antibodies among blood donors [18, 20]. A Among which one is conducted at Kassala Teaching Hospital, Eastern Sudan, HIV prevalence was determined as 3% [18]. The other study was conducted toward blood donors attending Kosti Teaching Hospital in the White Nile State. Over a period of 4 months (January 2014 to April 2014) a total of 1204 donations were processed. There were only 8 seropositive donors for HIV (0.7%) [20]. The result of meta-analysis (Fig. 2) showed overall pooled prevalence of 1.8% (95% CI -0.47 to 4.03), the heterogeneity was I^2^ = 92%.

Seven studies were found determining the prevalence of HBV among blood donors in Sudan [18, 20, 24, 27, 28, 35, 36]. Among which two studies were conducted in Khartoum [28, 36], two in Nyala, South Darfur State, Western Sudan [24, 35], one in Kassala, Eastern Sudan [18], one study in Elobeid [27] and one in Kosti [20]. One study [36] was found concerned with the prevalence of occult hepatitis B infection therefore no determination about the HBsAg was found. Moreover, another study [35] only present data of prevalence of co-infection of HBV and HCV among participants, these studies will be reviewed in their corresponding sections. The prevalence described in the remaining five studies ranges from 3.5 to 15.8%. The pooled prevalence was 6.9% (95% CI 4.89 to 8.97), the heterogeneity was I^2^ = 82%.

Four studies were identified providing prevalence data of HCV antibodies among blood donors in Sudan [18, 20, 24, 27], two studies were carried out in Elobeid and Kosti cities reporting prevalence of (3.5%) and (3.4%), respectively [20, 27], the other two studies were conducted in Nyala, South Darfur State, Western Sudan [24], and Kassala, Eastern Sudan [18], the percent prevalence of the virus in the two studies was (0.6) and (3.1%), respectively. The pooled prevalence was 2.6% (95% CI 0.97 to 4.21), the heterogeneity was I^2^ = 88% (Fig. 4).

### Prevalence among pregnant women

Five studies reported HIV prevalence among pregnant women in Sudan, one study was conducted in Kassala, Eastern Sudan [17], three in Khartoum State [11, 12, 14], and one in Gadarif State [15]. The prevalence rates ranged from 0 to 1.4%. The result of meta-analysis showed overall pooled prevalence of 0.4% (95% CI 0.14 to 0.66), the heterogeneity was I^2^ = 56%.

Two studies have been found concerned of the prevalence of HBsAg among pregnant women in Sudan; Elsheikh and colleagues’ study was a cross sectional conducted at Omdurman maternity hospital in Khartoum State in 2007 [26]. HBsAg was detected in 41 (5.6%) out of 728 tested women. Later on, in 2014, 20 (5.1%) out of 396 tested pregnant women were concluded to be positive for HBsAg in the study of Osman and colleagues [12]. The pooled prevalence was 5.5% (95% CI 4.13 to 6.80), the heterogeneity was I^2^ = 0%.

The same two studies have been identified establishing the prevalence of HCV antibodies among pregnant women. Elsheikh and colleagues reported low HCV prevalence of (0.6%) among pregnant women in Khartoum [26].While a higher prevalence of (1.3%) has been estimated in Wad Madani city among the same population [12]. The pooled prevalence was 0.6% (95% CI 0.28 to 1.03), with heterogeneity value of 0%.

### Prevalence among groups at risk

Five studies provided information on HIV antibodies prevalence among different populations at risk in Sudan [10, 13, 16, 19, 21]. Among which one relatively old study (conducted in 1997) focused in determining HIV antibodies among children (< 16 years) of Khartoum. HIV seroprevalence rate of 1.2% was determined [21]. In the same context, in Omdurman Teaching Hospital, Khartoum State in 2010 Abbas and colleagues determined the HIV antibodies prevalence of 5.7% (95% CI 2.1–11.9) in acutely hospitalized children aged 1.5–14 years [13]. Two studies were toward female sex workers (FSW) [10, 19]. One is conducted in Khartoum State in 2010 on 321 participants aged 18–49 year old; prevalence of 0.9% (95% CI 0.1–2.2) was determined [19]. The other study conducted by Elhadi and colleagues [10] in 2011–2012 was established in the capital cities of 14 States in Sudan. Their study classified Sudan into five large zones among which the fourteen sites have been distributed. Prevalence was provided for each site separately, and no further information was provided regarding specific State or city location of the sites despite of being in the zone (Western zone for instance). The highest HIV prevalence in their study was 5.0% and 7.7% as found in two sites located in the Eastern zone (Eastern Sudan), while in the other zones it ranged from 0 to 1.5%. The pooled prevalence of the fourteen prevalence rates alongside the prevalence determined in the study of Abdelrahim and colleagues was 0.8% (95% CI 0.45 to 1.10), the heterogeneity was I^2^ = 96%.

One study was conducted in Kassala Hospital, Eastern Sudan in 2012 provided HIV prevalence rate among newly infected TB patients with age ranged from 18 to 62 years, prevalence was determined as 18.3% [16].

Four studies were found describing the prevalence of HBsAg among different populations at risk in Sudan [22, 25, 29, 32]. The four studies were concerned about one of the following risk factors (HIV patients, hemodialysis patients, health workers, children with cancer).

The study of Yousif and colleagues [32] was conducted in Khartoum State in 2014; it was toward 358 HIV-positive treatment naive patients. Out of all HIV positive patients participating in their study; 96 (26.8%) were positive for HBV DNA using RT-PCR.

Mohammed and colleagues [29] conducted a cross-sectional study in the period from 2012 to 2014 to investigate the prevalence of HBsAg as well as occult hepatitis B among 100 haemodialysis patients, their study was conducted at three hospitals (Bashir Hospital, El Amel Hospital and Dr. Salma Center for Transplantation and Haemodialysis) in Khartoum State. (9%) of participants showed HBsAg positive results.

Elmukashfi and colleagues [25] conducted an observational, cross sectional study to determine the prevalence of HBsAg among healthcare workers in different Public Teaching hospitals in Khartoum State. It was carried out on stratified two-stage cluster random sampling of 843 healthcare workers. The prevalence among medical staff in their study was reported to be (6%). Abdalla and Hamad in their study in 2016 determined the prevalence of HBsAg among children with cancer in Sudan. The study was conducted at the Radioisotope Center in Khartoum. One hundred Seventy-eight children were enrolled in their study and (21.3%) showed HBsAg positive results [22].

Regarding HCV; three studies were found determining the prevalence of HCV antibodies among different populations at risk in Sudan [10, 31, 34]. In 2007 a cross sectional survey of patients on maintenance hemodialysis was conducted in Khartoum. The study reported a prevalence rate of the antibodies of the virus as 23.7% [34]. At the same year Mudawi and colleagues conducted a study on HIV-infected patients for HCV antibodies detection, the prevalence was determined to be 1.7% [31]. The study of Elhadi and colleagues conducted HCV seroprevalence surveys in ten sites of the total fourteen sites mentioned above while targeting the same population (Female Sex Workers). The prevalence rates ranged from 0 to 5.2%. The ten sites’ pooled prevalence was 1.6% (95% CI 0.56 to 2.65), with heterogeneity value of 88%.

### Prevalence related to demographic characteristics

In 2010, Abdalrahim and colleagues in their study on female sex workers determined that the majority (67.9%) was younger than 28 years and only (23.5%) had no education at all. Almost all respondents were Sudanese and Muslims, 99.7% and 90.2%, respectively [19]. In 2013, Elhadi and colleagues reported that almost (30%) of two sites (among the Western and Northern zones) had a comprehensive knowledge of HIV, while it was below 10% at five other sites [10]. In 2016, Adam and colleagues determined that there is a significant association of HIV infection and residence as the majority of HIV infected pregnant women were of urban residence (69.2%) [15].

Regarding HBV infection; the study of Abou and colleagues in 2009 reported that the highest percentage of HBV reacted samples were from participants aged within the age group 19–24 and 37–42 years old as (30.8%) for each [24]. Moreover, one study determined a significant relationship between seropositive HBsAg and participants between 20 and 40 years old [20], another study determined that more than half (57%) of those have HBsAg seropositive belong to the age group 28–37 years [27].

The study of Abdallah and colleagues determined a prevalence of (22%) among Rashiada tribe in Eastern Sudan [23]. Moreover, the same study determined statistical significant differences in residence, gender, age, illiteracy, and blood transfusion between seropositive and seronegative participants. The study of Elmukashfi and colleagues conducted a multicenter study in all federal as well as state teaching hospitals in Khartoum State and concluded that HBV is highly prevalent among healthcare workers in public teaching hospitals in Khartoum State [25]. Moreover, vaginal delivery, parity and home delivery were found to be associated with HBsAg seropostivitiy in the study of Osman and colleagues [12]. Abou and colleagues in their study concluded that the predisposing risk factors of HBV are unprotected sexual activities as the most apparent predisposing risk factor (20%), followed by razor sharing (13.3%), parenteral drug injections (10%), living in Egypt for long period and alcoholism (6.6%) for each, tattooing and surgical procedures (3.3%) for each [24], these risk factors were also determined in their study as risk factors for HCV infection as well.

Moreover, regarding the prevalence of HCV antibodies; Elzouki and colleagues reported that the virus rate was highly significant among population over 30 years [34]. Furthermore, a study in Nyala estimated that 50% increase in the prevalence is observed in the population belongs to the age range of (31–36) years old [24], another study determined that more than half (57%) of hepatitis C seropositive participants belong to the age group 28–37 years old [16]. Duration of dialysis, renal transplantation, dialysis in multiple centers and previous surgery have been reported as risk factors of HCV infection [34].

### Prevalence by region

**Fig. 5:**
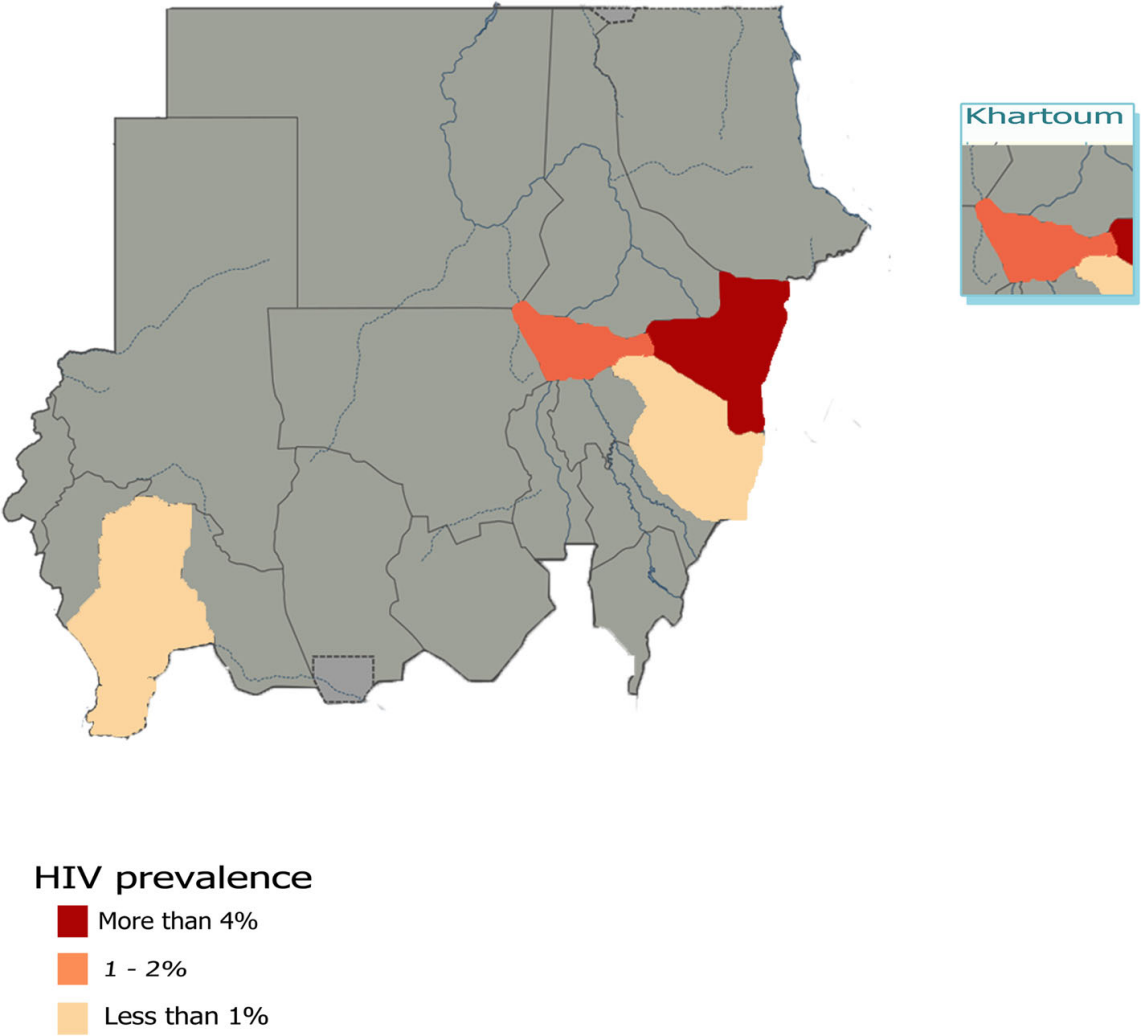
Prevalence of HIV antibodies in all corresponding States of Sudan from studies included in the review. Map only has information in States where included studies are conducted; all States where no included studies are available were shaded gray

Regarding HIV; six prevalence studies were conducted in Khartoum State; the most representative State [11–14, 19, 21], three in Kassala State [16–18] and two studies were conducted one in Gadarif [15], and one in Kosti [20]. The national study of Elhadi and colleagues [10] was not introduced here as the precise State names in all fourteen sites were not determined in their study. The highest percent prevalence of the virus (18.3) has been estimated in Kassala State in which the study was carried out among 109 participants [16]. Another study conducted in Kassala as well reported the lowest percentage of (0.2%) among 430 participants [17]. The third study in Kassala provided a prevalence of 3%. The pooled prevalence of all three studies was 4.2% (95% CI 0.61 to 7.74), the heterogeneity was high (I^2^ = 95%. The studies in Khartoum State provided a prevalence rates range from 0 to 5.7%. The pooled prevalence was 1.2% (95% CI 0.76 to 1.72), the heterogeneity was I^2^ = 18%. The only study conducted in Gadarif State recruited 6420 participants with HIV antibodies prevalence rate of 0.4% [15]. The study conducted in Kosti city has determined percent prevalence of 0.7% among study population of 1204 [20]. (Fig. 5) illustrates the prevalence of HIV antibodies in all corresponding States of Sudan from studies included in the review.

**Fig. 6:**
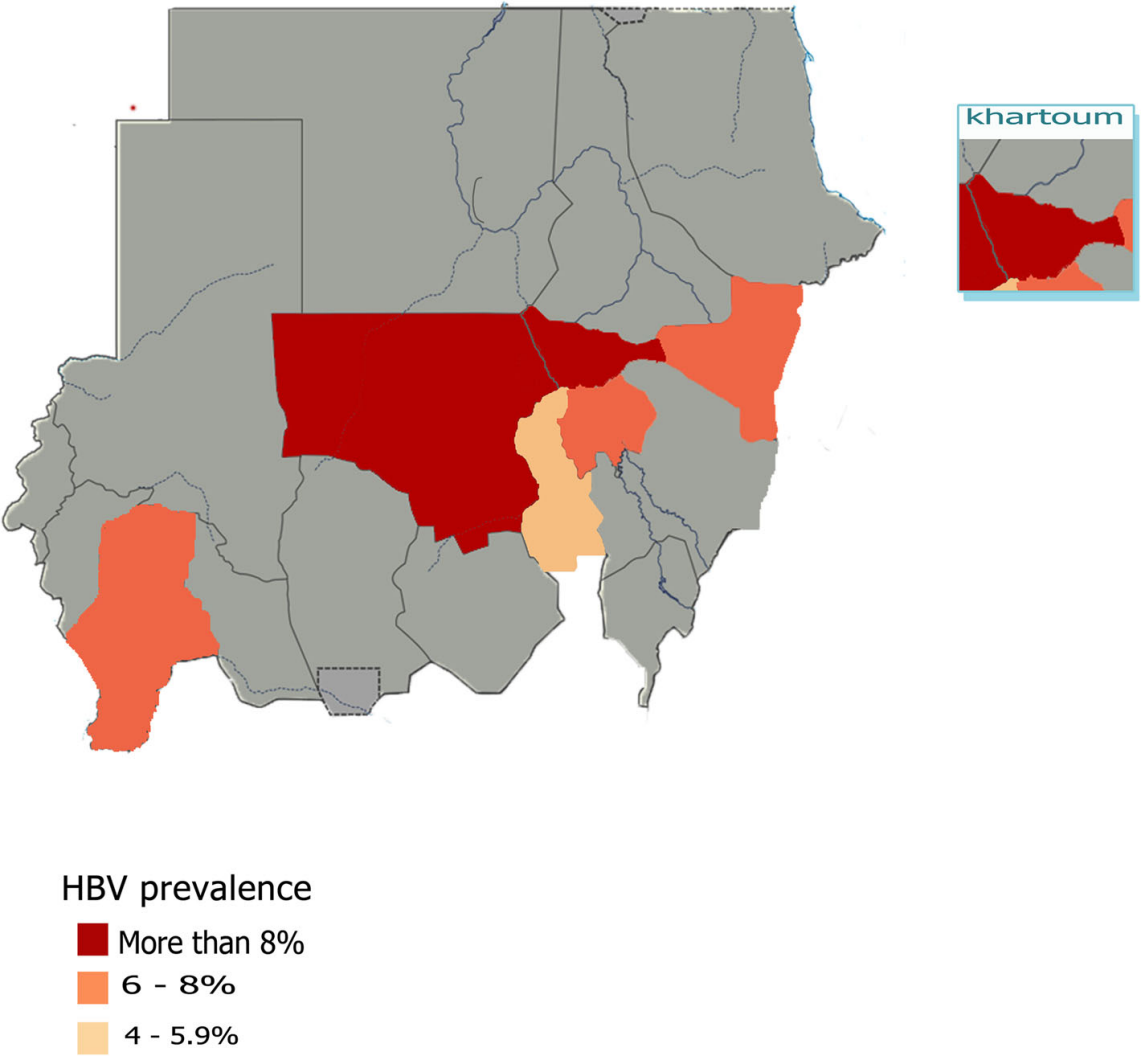
Prevalence of HBsAg seropositivity in all corresponding States of Sudan from studies included in the review. Map only has information in States where included studies are conducted; all States where no included studies are available were shaded gray

Eieght studies presented HBsAg prevalence rates for the Khartoum State; the most representative State [12, 22, 25, 26, 28, 29, 31, 32]. Among these studies, the HBV prevalence rates ranged from 5.1 to 26.8%. These studies presented HBV prevalence based on a total population of 3308. The pooled prevalence was 12.69% (95% CI 8.16 to 17.22), the heterogeneity was high (I^2^ = 95%). Only two studies were based in Kassala State, Eastern Sudan, the two studies represent a population of 1186, while prevalence rates reported as 8.2% and 4.3% [18, 23]. The pooled prevalence was 6.2% (95% CI 2.36 to 10), the heterogeneity was high (I^2^ = 90%). Wad Madani in Gezira State participated by two studies [12, 30], the studies presented HBV prevalence based on a total population of 404 and 285 participants, with prevalence rates of 6.8% and 5.1%, respectively. The pooled prevalence was 6% (95% CI 4.29 to 7.75) with 0% heterogeneity. Nyala in Southern Darfur participated with one study, the study presented HBV prevalence based on a total population of 400, with prevalence rate of 6.2% [24]. Kosti and Elobeid participated with one study per each as well, with total study population of 1204 and 400 with prevalence rates of 5.5% and 9.3%, respectively [20, 27] (Fig. 6) illustrates the prevalence of HBsAg in all corresponding States of Sudan from studies included in the review.

**Fig. 7:**
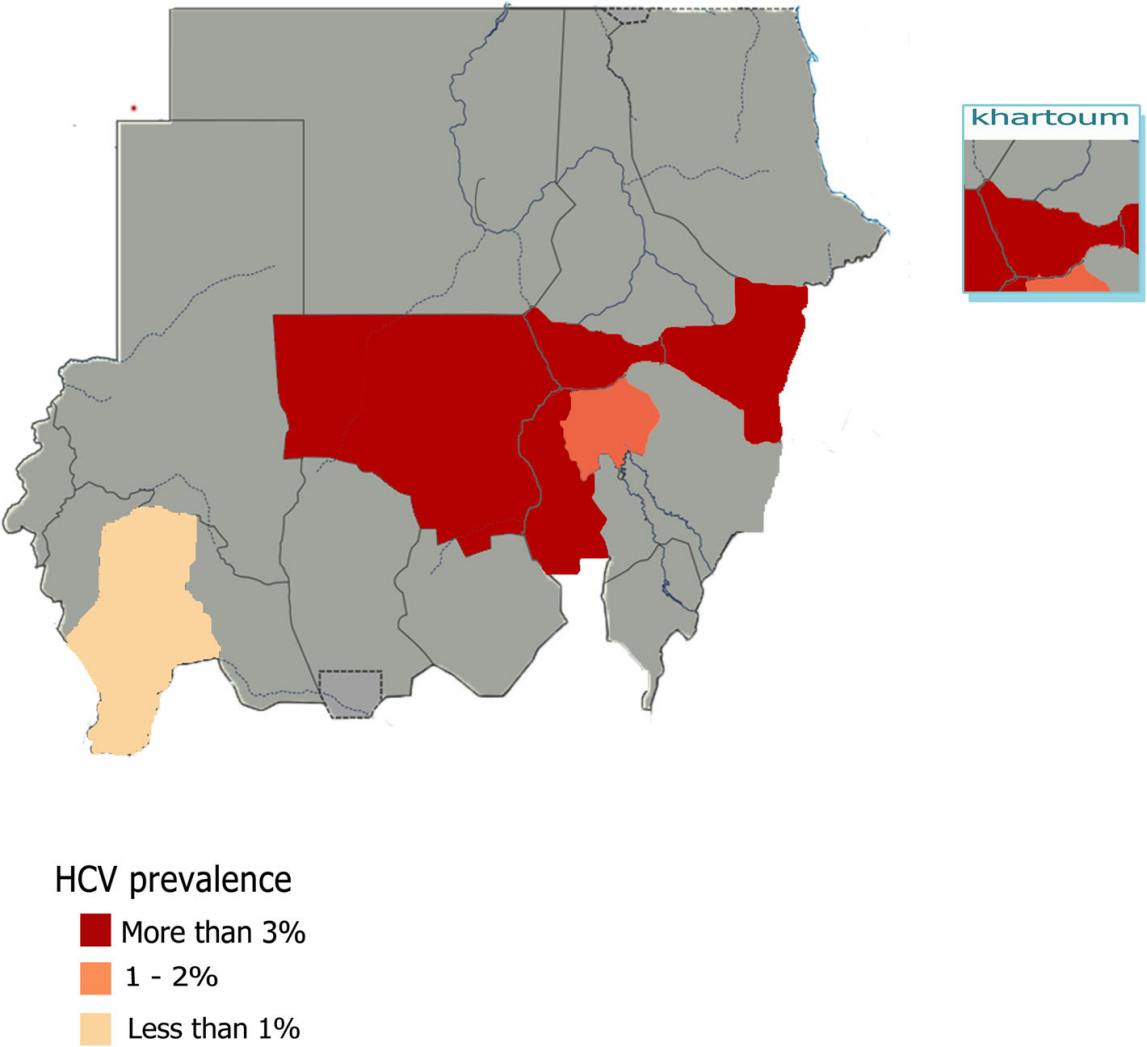
Prevalence of HCV antibodies in all corresponding States of Sudan from studies included in the review. Map only has information in States where included studies are conducted; all States where no included studies are available were shaded gray

Among the nine studies included regarding HCV antibodies prevalence; four were conducted in Khartoum State; the most representative State, while the remaining studies were conducted one in each of Wad Madani, Elobied, Kassala, Nyala and Kosti cities [12, 18, 20, 24, 27]. The highest percent prevalence of the virus (23.7%) has been estimated in Khartoum State in which a cross sectional study carried out among only 236 participants [34]. In contrast to a study conducted in Nyala that reported the lowest percentage of (0.6%) among 400 participants [24]. Also in Khartoum State two more studies have been established among relatively large populations 728 and 385 participants, nevertheless, they concluded lower prevalence rates of 0.6% and 1.7%, respectively [26, 31]. The pooled prevalence in Khartoum State was 6.8% (95% CI 2.26 to 11.29), with high heterogeneity value of 97%. Moreover, Elobied, Kassala and Kosti have determined percent prevalence above 3; (3.5%), (3.1%) and (3.4%), among populations of 400, 810 and 1204, respectively [18, 20, 27]. lastly, one study conducted in Wad Madani, Gezira State has reported that five (1.3%) out of 285 participants were seropositive for HCV antibodies [12]. (Fig. 7) illustrates the prevalence of HCV antibodies in all corresponding States of Sudan from studies included in the review.

### Prevalence by studies’ publication time

Regarding HIV; the earliest study among studies included in the review has been found to be published in 1997 with prevalence rate of 1.2% [21], followed by one study in 2006 with prevalence rate of 1% [11], In 2010, two studies were published with prevalence rates of 5.7% and 0.9% [13, 19]. The pooled prevalence was 2.8% (95% CI -1.79 to 7.42), the heterogeneity was I^2^ = 78%. In 2011 one study as well reported prevalence rate of 0.2% [17]. Followed by another two studies in 2012 with prevalence data of 18.3% and 3% [16, 18]. The pooled prevalence was 10.2% (95% CI 4.76 to 25.18), the heterogeneity was high (I^2^ = 94%). In 2013, the multi-centre national study of Elhadi and colleagues was conducted with prevalence rate of 0.8%. Furthermore, one study was published in 2014 with prevalence rate of 1.5% [10]. The next two years witnessed publication of two studies one in 2015 with prevalence rate 0.7% [20], and the other one in 2016 with prevalence rate of 0.4% [15].

The earliest studies among studies included in the review and concerned of the prevalence of HBsAg has been found to be published in 2007 with prevalence rates of 5.6% and 6.2% [26, 30]. The pooled prevalence was 6% (95% CI 4.66 to 7.27), the heterogeneity was I^2^ = 0%. Followed by one study providing HBsAg prevalence rate of 6.2% in 2009 [24], in 2011, two studies were published with prevalence data of 8.2% and 11% [23, 28], the pooled prevalence was 9.4% (95% CI 6.67 to 12.09), the heterogeneity was I^2^ = 59%. Another two studies follow in 2012 with prevalence data of 4.3% and 6% [18, 25]. The pooled prevalence was 5.1% (95% CI 3.44 to 6.77), the heterogeneity was I^2^ = 61%. In 2014, three studies were published providing a prevalence data ranges from 5.1 up to 26.81% [12, 31, 32]. The pooled prevalence was 14.4% (95% CI 3.06 to 25.75), the heterogeneity was high (I^2^ = 97%). Two studies were published in 2015 with prevalence rates of 5.5% and 9% [27, 29] providing a pooled prevalence value of 8.3% (95% CI 3.22 to 13.45), the heterogeneity was I^2^ = 0%. Two studies were published in 2016 providing prevalence data of 21.3% and 9.3% [22, 27], Pooled prevalence was 15.02% (95% CI 3.27 to 26.77), the heterogeneity was I^2^ = 92%.

**Fig. 8:**
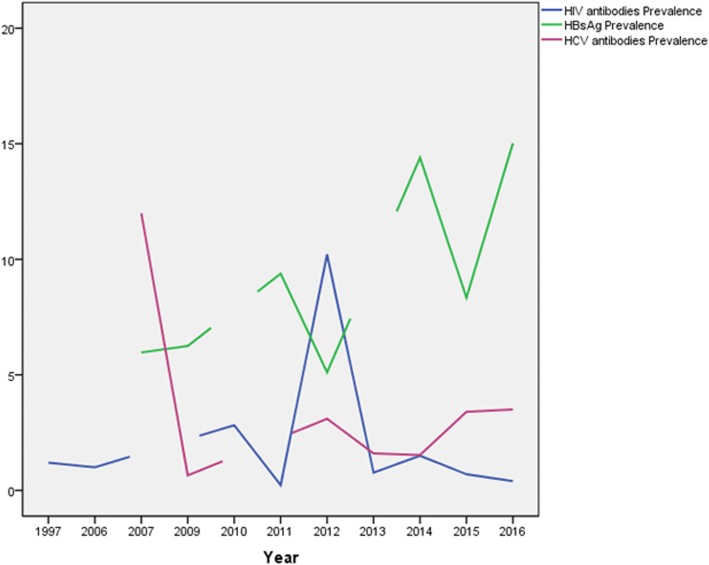
Prevalence rates of the three infections according to publication period of all studies included in the review. Data was plotted as line graphs, when there is no published study in a given year; the line predicts the direction to be in accordance to the next year with published study/ies and get disrupted to indicate a missing data

For HCV; the nine research articles included in this review have been established in the period of ten years; from 2007 up to 2016. The earliest studies were carried out in 2007 reporting prevalence rates of 0.6% and the highest measure of 23.7% [26, 34]. The pooled prevalence was 11.9 (95% CI -10.64 to 34.63) the heterogeneity was I^2^ = 99%. In 2009 Abou and colleagues conducted a study in Nyala that reported a prevalence of 0.65% [24]. Moreover, a percent prevalence of 3.1 was reported in 2012 [18]. The national survey conducted by Elhadi and colleagues was conducted in 2013, and the ten sites’ pooled prevalence was 1.6% (95% CI 0.56 to 2.65) [10]. Furthermore, two studies were published in 2014 estimating prevalence rates of 1.3% and 1.7% [12, 31]. The pooled prevalence was 1.5% (95% CI 0.64 to 2.42), with heterogeneity value of 0%. The percent prevalence of the virus in 2015 and 2016 were 3.4 and 3.5, respectively [20, 27]. (Fig. 8) illustrates the prevalence of the rates of the three infections according to publication period.

### Occult hepatitis B

Five different eligible studies were found determining the prevalence of occult hepatitis B infection among different study populations [28, 29, 31, 32, 36]. The study of Mudawi and colleagues in 2014 determined the OBI prevalence among HIV infected patients in Khartoum State to be (15.1%) [31]. Moreover, the same research group conducted a similar study applying the same study sitting later in the same year determining the prevalence to be even higher (20%) [32]. Mohammed and colleagues in the same year provided a prevalence of OBI among a different risk group (hemodialysis patients), participants were enrolled in three consecutive years from different health facilities in Khartoum State and the prevalence was determined as (3.3%) [29]. Mahgoub and colleagues in 2010 conducted a study to determine the prevalence of HBsAg and OBI among 404 blood donors from Khartoum State, OBI prevalence was determined as (4.6%) [28]. One more study was conducted by Mahmoud and colleagues among blood donors as well in Khartoum State, the study sitting was planned to detect several HBV genes (S, C and X) as well as to quantify DNA. As described by authors, genes were present in more than 35% of the participants. However, HBV DNA was not detected in any HBsAg negative sample [36]. The overall pooled prevalence of OBI is 12.3% (95% CI 4 to 20.68), the heterogeneity was high (I^2^ = 99%).

### Prevalence of HIV, HBV and HCV co-infection

Three studies was found determining co-infection prevalence of two viruses in the same population. The study of Osman and colleagues conducted a cross sectional study in pregnant women in Wad Madani city, Gezira State in 2014 and determined that (0.3% 1/285) of women participated in their study was seropositive for both HBV and HCV [12]. Moreover, Abou and Eltahir provided co-infection data among blood donors in Nyala city in 2009, the seropositivity of HBV and HCV dual infection was detected in only one participant as well (0.2% 1/400) [35]. Furthermore, a retrospective study targeting blood donors in Kassala Teaching Hospital, Eastern Sudan was conducted, co-infection of HBV and HCV was reported to be the most type of combination (6\810, 0.74%) followed by HBV and HIV (0.6% 5\810). Co-infection of both HIV and HCV was also reported in one participant (0.1% 1\810) [18].

## Discussion

The current evidence regarding prevalence of HIV, HBV and HCV infections among general population in Sudan is in need of enforcement. Determining prevalence estimates for all three viruses is crucial for establishing appropriate country specific strategies regarding prevention, diagnosis, and containment. Forty nine studies of seroprevalence targeting the three viruses were - for the first time - quantitatively analyzed to better determine the burden of these infections in the country.

HIV surveillance in Sudan in particular has been described to be of good function according to the assessment of the quality of HIV surveillance in low and middle-income countries [37]. All included studies determined HIV antibodies prevalence range of 0 to 18.3% among different study populations, three studies concluded a prevalence more than 3% - which is the threshold of HIV infection to be considered as high [38], however, meta-analysis showed overall pooled prevalence of 1%. This general prevalence estimate is low when compared to the prevalence reported from some neighboring countries; as it was estimated in South Sudan as 3.4%, and Djibouti as 2.1%, data from Somalia indicate almost similar HIV prevalence as 1.1% [39].

.This result does not agree to the international estimates of HIV in 2011 as they indicate HIV prevalence of 0.5% among adults in Sudan [40]. However, the fact that this international estimate was almost six years ago is needed to be considered.

HIV pooled prevalence among pregnant women in Sudan was estimated to be 0.4%, which is higher when compared to prevalence data among pregnant women in Morocco (0.2%), and Iran (zero prevalence) [39]. The pooled prevalence of HIV among female sex workers is showed to be 0.8% according to the national study conducted in fourteen States in Sudan by Elhadi and colleagues [10]. United States reported prevalence rates among the same population to be from 0.3% up to 32.1% according to the recently (2016) published systematic review, the pooled prevalence was 17.3% (95% CI 13.5–21.9%) [41]. Moreover, HIV prevalence ranged between 5.4 and 7.4% among Female Sex Workers in Iran [42].

Only one study conducted at Kassala, Eastern Sudan in 2012 provided HIV prevalence rate among TB patients, prevalence was determined as 18.3% [16]. This finding is alarming as it showed a high burden of HIV among TB patients in Sudan, as this rate is higher than many known prevalence rates; South Sudan reported a rate of 14.7%, followed by Djibouti 11.3%, Somalia 8.2%, Iran 3.8%, Yemen 1.6%, Morocco 0.8%, 0.4% in Saudi Arabia and zero was reported in both Palestine and Jordan [39]. However, the fact that the study conducted in Kassala only had 109 TB participants underestimate the significant of this finding.

For HBV, four studies out of the fourteen studies reported prevalence higher than 8%, the pooled prevalence was 9.1% (95% CI 7.11 to 11.04). This prevalence rate is considered very high when compared to the most endemic country in Europe (Italy) which is reported to have prevalence rate of 5.9% as determined by a recently published systematic review in Europe (2016) [43]. Moreover, according to WHO, this prevalence rate is high (≥8%). This prevalence is with an accordance with the reported prevalence in Africa (8.8%) in the recently published systematic review estimating the worldwide prevalence of hepatitis B virus infection [44]. Moreover, the same systematic review indicated HBsAg prevalence in Sudan in particular as 9.8 (95% CI 9.03 to 10.54). On the other hand, this estimated prevalence is lower than the prevalence reported in Cameroon as the overall prevalence of HBV infection among 105,601 participants was determined as 11.2% (95% CI 9.7% to 12.8%) [45].

The rates in the Middle East do vary as reported in the study of Gasim in 2013; a range from 0.6 to 8% in different countries was reported. 5.1% in Yemen, 4.2% in Saudi Arabia [46], 3.6% in Algeria, 3.5% in Kuwait and Palestine, and 2.1% in Iran [47].

Close to half of the studies (44%) estimated rate of HCV antibodies prevalence above 2% - the level at which anti-HCV prevalence rate is considered to be high [48]. The overall pooled prevalence was 2.5% (95% CI 1.42 to 3.53). In developed countries like the US, prevalence of HCV infection has been estimated to be less than 2% [49]. Even in the Middle East, two recent systematic reviews (2017, 2018) conducted in Iran indicated that the overall seroprevalence of HCV in the general population is 0.6% and 0.3%, respectively [50, 51], 0.3% is reported in Bahrain, 0.4% in Oman, 1.1% in Qatar, 1.4% in Kuwait, 1.6% in Saudi Arabia and United Arab Emirates [52] and it is estimated as 1% in Turkey [53]. This may highlight that the level of HCV infection in Sudan may be relatively high. Nevertheless, this prevalence rate is lower when compared to several countries; recently published systematic reviews concluded HCV antibodies prevalence rate of 3% and 5.9% in Ghana and Italy, respectively [43, 54]. Moreover, this prevalence rate is much lower when compared to the north neighboring country. Egypt is encountered with a huge HCV infection; it is reported to have a prevalence rate of 14.7% and 11.9% in recently published reviews (2016, 2018, respectively) [55, 56].

The prevalence of HCV antibodies among pregnant women was estimated as 2.6%, this is considered lower when compared to the prevalence in Ghana 4.6% [54] and relatively higher or comparable - to some extent - when reference is made to regions like the United States and Europe as general where prevalence of chronic HCV among this group has been estimated to be around 1–2.5% [57]. The high HCV prevalence among pregnant women emphasizes the need for the adoption of a national program that includes HCV screening for pregnant women at risk [57].

Only one study; Mudawi and colleagues’ study in 2014 retrieved 358 HIV-infected patients for HCV antibodies detection, the prevalence is estimated to be 1.7% [31]. Compared to the pooled prevalence conducted in Ghana; this result is lower as it is determined as 2.8% (95% CI = 0.4–6%) [54]. Furthermore, it is considered very low when compared to the prevalence measure reported in a very recent systematic review from Iran (2018) as HCV antibodies prevalence among HIV-infected patients was determined as 67% [51]. However, it is to be noted that the systematic review conducted in Iran [51] included 25 studies concerned of the prevalence of HCV antibodies among HIV-infected patients while only one study was included in the current systematic review.

Two included studies reported a prevalence rate ranges from 15.1 to 20% of OBI among HIV infected patients in Sudan [31, 32]. This prevalence is higher when compared to the study of Rajat and colleagues in New York in 2013 as they reported a prevalence of 7% [58]. Moreover, one study determined the prevalence of OBI among hemodialysis patients; group is known to be of high risk, the prevalence was determined as 3.3%, which is higher than a prevalence reported in a study conducted in Iran (0%) among 400 hemodialysis patients [59]. Nevertheless, it is to be considered that included studies that addressed OBI is scarce to conduct a good quality assessment as only 5 out of 14 included studies concerned of the prevalence of HBV prevalence were provided OBI information. As a result; those differences may be attributed to demographics or sample size of participants.

Based on data reviewed and synthesized; there is no evidence for an HIV endemic in the general population of Sudan. However, both HBV and HCV seroprevalence rates are indicating otherwise. It is to be emphasized that studies toward determining prevalence measures among potential bridging populations (such as truck drivers and military personnel), tea sellers, Men who have Sex with Men (MSM), whip battered individuals and drug users were not included in this review. It is well known that these populations generally express risky behavior.

Several risk factors are to be analyzed to help in firstly understand the situation, then controlling the current critical burden of HBV and HCV consequently. The neighboring Egypt is confronted with a huge HCV infection problem, it has the highest prevalence of HCV in the world, and HCV infection and its complications are among the leading public health threats as Estes and colleagues recently reported (2015) [60]. To complete the picture; since the 1990s, a new inflow of refugees started arriving in Egypt as a result of wars in the area, especially Sudan, Ethiopia, Eritrea and Somalia. According to official statistics, around 32,000 are granted refugee status in Egypt, among those, 73% are Sudanese [61]. This situation is overcritical as not only Egyptian as well as Sudanese regular travelers may act as a possible threat of increasing HCV prevalence in Sudan, but both returning legal and illegal Sudanese refugees. For HBV; it is estimated that more than 6% (more than 5 millions) are HBsAg seropositive in Ethiopia and more than 22% (more than 2 millions) are HBsAg seropositive in South Sudan [44]. Not controlled movements through the Sudanese borders of these countries will have its consequences in the national prevalence of HBV and HCV. Regarding another potential threat from inside; Sudan is reported among other countries in the region to have the highest number of needle-stick-acquired infections (due to mostly lack of sterilization). As a matter of fact, injection practices are reported to be much safer in several countries in the sub-Saharan Africa [62].

Prevention against HBV infection can be adopted by increasing the distribution of the vaccine, especially in rural areas and in populations at risk. Moreover, when understanding that many people at risk may not know the possible routes of transmission of these viruses or behave in indifference manners due to lack of awareness or social stigma, it is recommended that people at risk should be vaccinated free of charge. Unfortunately, unlike HBV, vaccines for HIV and HCV infections are currently unavailable and therefore disruption of infection transmission would rely primarily on education to improve knowledge and awareness of the transmission dynamics of the viruses. To sum up, reducing the overall burden of HIV, HBV and HCV infections in Sudan will require new measures and national strategies and the recognition of the infections as one of the country’s priority issues.

## Strengths and limitations

The strengths of this review are that we systematically identified and included prevalence estimates without specific time limits, all studies with good quality were enrolled despite of the publication time. Moreover, we have conducted meta-analysis to derive a pooled prevalence estimate of all included prevalence studies. Furthermore, we carried out a quality assessment of the included studies based on criteria specifically developed to determine the quality and the degree of selection bias in the studied populations.

Nevertheless, several limitations are to be considered when interpreting study results; gray literature evidence was not assessed, although all included studies are of good quality, several good studies might have been missed. Furthermore, another parameter that should be considered is that the limited number of studies in some States can be noticed for which the outcome might not be suitable to be generalized to the State level. Moreover, the low sample population in general is another limiting factor, HIV, HBV and HCV seroprevalence was determined according to the recruitment of (15,947), (5948) and (8643) participants, respectively.

## Conclusion

The current study indicates HIV sreoprevalence status below the endemic level. However, both HBV and HCV infections are considered endemic in Sudan according to the current evidence. Different prevalence rates were observed in different States, this difference itself might highlight the possibility for containment and control. The current study is of low sample size when considering the whole population and hence there is a need to conduct larger studies to monitor the burden of these deadly infections.

## Additional file

Additional file 1: **Table S1.** PRISMA checklist. **Table S2.** Quality assessment of all included studies. **Table S3.** Summary of studies included in the review. (DOCX 60 kb)
